# The Beneficial Role of *Filipendula ulmaria* Extract in Prevention of Prodepressant Effect and Cognitive Impairment Induced by Nanoparticles of Calcium Phosphates in Rats

**DOI:** 10.1155/2021/6670135

**Published:** 2021-02-10

**Authors:** Natalija Arsenijevic, Dragica Selakovic, Jelena S. Katanic Stankovic, Vladimir Mihailovic, Slobodanka Mitrovic, Jovana Milenkovic, Pavle Milanovic, Miroslav Vasovic, Snezana D. Markovic, Marko Zivanovic, Jelena Grujic, Nemanja Jovicic, Gvozden Rosic

**Affiliations:** ^1^Department of Dentistry, Faculty of Medical Sciences, University of Kragujevac, 34000 Kragujevac, Serbia; ^2^Department of Physiology, Faculty of Medical Sciences, University of Kragujevac, 34000 Kragujevac, Serbia; ^3^Department of Science, Institute for Information Technologies Kragujevac, University of Kragujevac, 34000 Kragujevac, Serbia; ^4^Department of Chemistry, Faculty of Science, University of Kragujevac, 34000 Kragujevac, Serbia; ^5^Department of Pathology, Faculty of Medical Sciences, University of Kragujevac, 34000 Kragujevac, Serbia; ^6^Department for Biology and Ecology, Faculty of Science, University of Kragujevac, 34000 Kragujevac, Serbia; ^7^BioIRC, Bioengineering R&D Center, 34000 Kragujevac, Serbia; ^8^Department of Histology and Embryology, Faculty of Medical Sciences, University of Kragujevac, 34000 Kragujevac, Serbia

## Abstract

Mineral components of dental composites are used in many medical and dental applications, including preventive, restorative, and regenerative dentistry. To evaluate the behavioural alterations induced by nanosized particles of novel dental composites, by means of depressive level and cognitive functions, experimental groups of rats were chronically administered with nanosized hydroxyapatite (HA), tricalcium phosphate (TCP), and amorphous calcium phosphate (ACP) with or without simultaneous application of *Filipendula ulmaria* L. (FU) methanolic extract. The significant prodepressant action was observed in groups solely treated with HA and ACP. Besides, prolonged treatment with ACP also resulted in a significant decline in cognitive functions estimated in the novel object recognition test. The adverse impact of calcium phosphates on estimated behavioural functions was accompanied by increased oxidative damage and apoptotic markers in the prefrontal cortex, as well as diminished specific neurotrophin (BDNF) and gabaergic expression. The results of our investigation showed that simultaneous antioxidant supplementation with FU extract prevented calcium phosphate-induced behavioural disturbances, as well as prooxidative and apoptotic actions, with the simultaneous restoration of BDNF and GABA-A receptors in the prefrontal cortex. These findings suggest that FU may be useful in the prevention of prodepressant impact and cognitive decline as early as the manifestation of calcium phosphate-induced neurotoxicity.

## 1. Introduction

Calcium phosphate (CaP) compounds are widely used in preventive, restorative, and regenerative treatments in various fields of medicine [1]. The use of CaP compounds, naturally present in the process of bone maturation [2], has primarily shown importance in areas of the medical treatment and rehabilitation of bone tissues and, further, in the prevention and therapy of dental diseases.

Calcium phosphates are progressively used in restorative dentistry. The widespread clinical application of dental composites has indicated significant shortcomings of these materials. The most common cause for decay of modern composite fillings is the formation of secondary caries, followed by further loss of tooth tissue and the need for filling replacement [3]. Although secondary caries develops as a consequence of the imperfection of composite materials, the impact of the filling to the solid dental tissues is the most often passive. One way to prevent the recurrence of caries is to provide a high concentration of Ca^2+^ and PO_4_^3-^ dissolved in saliva. These ions originate from the mineral component of dental composites, which creates conditions for the remineralization of tissues that directly surround the filling [4]. There are only a few commercial preparations of dental composites with the ability to remineralize dental tissues. However, numerous studies are investigating different forms of CaPs (hydroxyapatite (HA), monocalcium phosphates, dicalcium phosphates, tricalcium phosphate (TCP), tetracalcium phosphate, and amorphous calcium phosphate (ACP)), as a bioactive component of composites [5]. The amorphous structure and chemical composition of ACP, the direct precursor of HA [6], cause the formation of high concentrations of ions in aqueous solutions [7]. In order to increase the remineralization potential, bioactive dental composites usually contain CaPs in the form of nanoparticles [8].

Although previous studies have confirmed the biocompatibility of these compounds, studies of nanosized CaP toxicity have yielded conflicting results [9]. Thus, Liu and colleagues indicated that systemic administration of nano-HA (in rabbits) caused liver function alterations manifested by increasing AST, ALT, and ALP levels [10]. Also, it was noted that the main mechanism of the nano-HA-induced osteoblast demise is apoptosis associated with ROS hyperproduction and mitochondrial, lysosome, and DNA damage [11]. Furthermore, the nano-TCP particles incorporated into rabbit tibial defects elicited an excessive and prolonged inflammatory response associated with decreased bone regeneration [12]. *In vitro* studies revealed that nano-TCP induced dose-dependent cytotoxicity, while nanoparticle transport via endocytosis was accompanied by an intracellular increase in calcium and phosphate ions, increased ROS production, and both external and internal pathway-mediated apoptosis [13]. Li and colleagues showed that ACP nanoparticles induced leukemia cell apoptosis by affecting the G1 phase of the cell cycle in particular, where the cytotoxic effect was directly dependent on ACP exposure interval [14].

On the other hand, literature data confirmed that the natural products with antioxidant effects, such as curcumin [15] and chicory extract [16], reduced the harmful effects of chronic CaP intake [17]. Among the natural products with a confirmed antioxidant action is the extract of *Filipendula ulmaria* [18]. *Filipendula ulmaria* (L.) Maxim. (*Rosaceae*) (FU), known as meadowsweet, is a perennial plant found in wild and cultivated habitats in Europe and Asia [19]. All parts of the plant are traditionally used for medical purposes, with the most beneficial effects observed from aerial parts [20]. FU has been used in the treatment of various inflammatory processes, rheumatism, neuralgia, diarrhea, and urinary tract infections [21]. A plethora of evidence of the numerous beneficial effects of FU is directly attributed to its specific polyphenolic content, rich in anti-inflammatory [20, 22], antioxidant [23, 24], and antimicrobial [24] compounds. Previous phytochemical investigations of *F. ulmaria* by our team [18] lead to a conclusion that some specific bioactive compounds determined in this extract, such as catechin, rutin, and spiraeoside [24], may be also responsible for antioxidant actions in the nervous system. Analyzing data concerning the distribution and mechanism of action for nano-CaPs after systemic application [25], the neurotoxic effects of CaP nanoparticles following this route of the administration still appear to be insufficiently elucidated. Although it has been reported that these particles cross the blood-brain barrier [26], their effect on the central nervous system is not fully investigated.

The use of nanosized CaP particles in medical practice, as the most potent in the prevention of damage and in regeneration of mineralized tissue, implicates the necessity to clarify the system toxicity, including neurotoxicity, as well as the potential ways to reduce their side effects. Following the findings that the principle mechanism of CaP toxicity involves oxidative imbalance, it seems reasonable to examine the antioxidant effect of FU, following experimental nanosized CaP prolonged load. Therefore, the aim of this study was to examine the beneficial effects of FU on changes in the cognitive functions and depressive behaviour, as the functional manifestations of nano-CaP-induced neurotoxicity.

## 2. Materials and Methods

### 2.1. Animals and Treatment

Two-month-old male Wistar rats, with b.w. range of 180–220 g, were obtained from the Military Medical Academy, Belgrade, Serbia. The animals were kept in the translucent cages in the groups of three under the standard conditions (temperature 23 ± 1°C, humidity 50 ± 5%) with light/dark cycle (12/12 h) and were provided standard chow and water *ad libitum* for the duration of the study.

**Figure 1:**
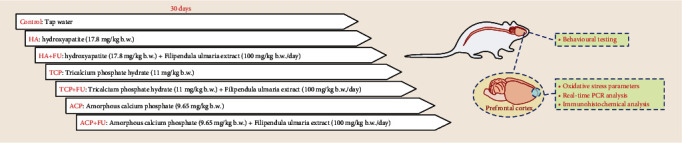
Experimental design.

The animals were randomised into seven equal groups (6 rats per group): control group; groups that orally received CaP nanoparticles in a daily dose of hydroxyapatite (17.8 mg/kg b.w.)—HA, tricalcium phosphate hydrate (11 mg/kg b.w.)—TCP, amorphous calcium phosphate (9.65 mg/kg b.w.)—ACP, solely, or simultaneously with *F. ulmaria* extract (100 mg/kg b.w.)—HA+FU, TCP+FU, and ACP+FU group. All protocols continuously lasted for 30 days, as presented in Figure 1.

The mineral components of dental composites in the nanoparticles were obtained from Sigma-Aldrich, Germany: hydroxyapatite nanopowder, <200 nm particle size (BET), ≥97%, synthetic; tricalcium phosphate hydrate nanopowder, <200 nm particle size (BET); calcium phosphate, amorphous nanopowder, <150 nm particle size (BET). The applied doses of mineral components were selected to be equimolar levelling the lowest dose of HA that showed toxic effects in the previously published study performed *in vivo* with nanoparticles in the rodents [27]. Also, the doses of CaPs in this study were chosen according to the reported release of mineral components from dental composites obtained *in vitro* [28]. The oral administration was chosen in order to mimic the authentic application route of investigated materials in the human population. The plant material (from the aerial part) and preparation of the extract as well as the content identification was performed using the previously described procedure [18]. FU extract dose was selected on the basis of our previous study that confirmed biological activity of this medicinal plant [18], reaching the optimal effective concentration at 100 mg/kg b.w. daily. The final concentration of all applied substances was calculated on the basis of an average water intake in the previous 24 h and dissolved in tap water.

All research procedures were carried out in accordance with the European Directive for the welfare of laboratory animals no. 86/609/EEC and the principles of Good Laboratory Practice and in accordance with the ARRIVE guidelines. All experiments were approved by the Ethical Committee of the Faculty of Medical Sciences, University of Kragujevac, Kragujevac, Serbia.

### 2.2. Behavioural Testing

Twenty-four hours after completing protocols, the animals were placed in the testing room for 1–2 h prior to the initiation of each test (approximately at 8 a.m.). All tests were performed under proper conditions of silence and illumination. During the trials, the experimenters were not present in the testing room. At the end of each trial, the experimental fields were cleaned in order to remove possible interfering scents. All tests were recorded by digital video camera mounted above the mazes at appropriate heights. Video files from the novel object recognition test were analyzed using EthoVision software (XT 12), an integrating video tracking system for automatic recording of activity movement and interactions of animals (Noldus Information Technology, the Netherlands).

#### 2.2.1. Tail Suspension Test

For the evaluation of depressive state level, we used the tail suspension test (TST). This test has been successfully used for that purpose for decades and was performed on the previously standardized procedure with described apparatus [29]. In TST, we determined the latency to the first immobility (s), the number of immobility episodes, the total duration of immobility (s), and an average duration of an immobility episode (s).

#### 2.2.2. Novel Object Recognition Test

Testing for the estimation of cognitive functions, the novel object recognition (NOR) test was performed according to the methods presented by Bevins and Besheer [30], with modification by Mumby and coworkers [31]. Testing involved three phases (performed in square arena 60 × 60 × 30 cm): phase 1—familiarization with the empty testing arena, phase 2—the introduction of two identical objects in the same arena placed on a fixed position, and phase 3—the replacement of one familiar object (from phase 2) with the new one (novel object). All phases lasted for five minutes, with intersession interval of 45 minutes. The following parameters were estimated (using EthoVision software XT12) in the NOR test: the frequency to novel object zone, the cumulative duration in novel object zone (s), and the time interval while head directed to novel object (s).

### 2.3. Determination of Tissue Oxidative Stress Parameters

Prefrontal cortex (PFC) homogenates were prepared according to the standard procedure previously established in our lab, as reported by Kumburovic and coworkers [32] and Vukovic and colleagues [33], with the chemicals listed in Supplementary file 2. Principally, phosphate-buffered saline (PBS, pH 7.4) was used for PFC tissue homogenization, and after centrifugation at 4000 rpm for 15 min at 4°C, the supernatant was separated and used for further analyses. For all spectrophotometric analyses, a UV-Vis double beam spectrophotometer (model Halo DB-20S, with a temperature controller, Dynamica GmbH, Dietikon, Switzerland) was used. Lowry's method was used for the evaluation of protein content in the homogenates, and bovine serum albumin was used as a standard [34]. The oxidative stress parameters tested in this study were the activities of superoxide dismutase (SOD) and catalase (CAT) as well as the levels of reduced glutathione (GSH) and thiobarbituric acid reactive substance (TBARS). The activity of SOD was determined according to the spectrophotometric method reported by Misra and Fridovich [35] where the inhibition of epinephrine transformation to adrenochrome was monitored. The CAT is known for its ability to catalyze the decomposition of hydrogen peroxide to water and oxygen which is, therefore, used in the method of Beers and Sizer [36] based on the rate of hydrogen peroxide degradation. The activities of both tested enzymes were expressed as units per milligram of proteins (U/mg). The level of GSH in tested samples was monitored according to Ellman's method [37] using 5,5-dithio-bis-(2-nitrobenzoic acid). The concentration of GSH in samples was expressed in milligrams GSH per gram of proteins (mg GSH/g). The products of lipid oxidation in tissue samples were estimated as the levels of thiobarbituric acid reactive substance (TBARS) and expressed as nmol of malondialdehyde (MDA) per milligram of proteins (nmol MDA/mg) [38].

### 2.4. Prefrontal Cortex RNA Isolation and Real-Time PCR Analysis

Total RNA was extracted from PFC tissue using PureZOL reagent (Bio-Rad, USA) according to the manufacturer's instructions. Reverse transcription was done using iScript Reverse Transcription Mastermix (Bio-Rad, USA). Quantitative RT-PCR was performed using SsoAdvanced Universal SYBR Green Supermix (Bio-Rad, USA). mRNA specific primers for Bax, Bcl-2, BDNF, anti-GABA A receptor alpha 5/GABRA5, and *β*-actin as a housekeeping gene were used (Supplementary file (available here)). Quantitative RT-PCR reactions were done in Applied Biosystems 7500 (Applied Biosystems, USA), and after data analysis, the relative gene expression was calculated according to Livak and Schmittgen [39].

### 2.5. Immunohistochemical Analysis

The prefrontal tissue specimens were fixed in 4% formaldehyde solution and embedded in paraffin. Coronal brain sections, 5 *μ*m thick, were dewaxed, rehydrated, and treated with citrate buffer (pH 6.0) in the microwave for antigen retrieval. Staining was visualized by using the EXPOSE Rabbit Specific HRP/DAB Detection IHC Kit (ab80437, Abcam, UK), and sections were counterstained with Mayer's hematoxylin. The slices were incubated with GABRA5 antibody (PA5-77413, Thermo Fisher Scientific, USA) and recombinant anti-BDNF antibody (EPR1292) (ab108319, Abcam, UK) overnight at room temperature. Sections were photomicrographed with a digital camera mounted on a light microscope (Olympus BX51, Japan), digitized and analyzed. The analyses were made by independent experimenters who were blind to the experimental protocol.

### 2.6. Statistical Analysis

Statistical analysis was performed with SPSS version 20.0 statistical package (IBM SPSS Statistics 20). The results are expressed as the means ± standard errors of the mean (S.E.M.). The parameters were initially submitted to Levene's test for homogeneity of variance and to Shapiro-Wilk test of normality. One-way ANOVA, followed by Bonferroni test, was used for comparisons between the groups. The significance was determined at *p* < 0.05 for all tests.

## 3. Results

**Figure 2:**
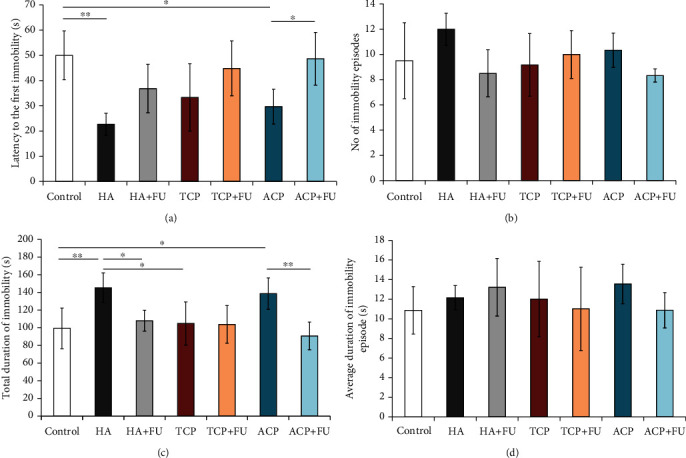
Parameters obtained in the tail suspension test: (a) the latency to the first immobility, (b) the number of immobility episodes, (c) the total duration of immobility, and (d) the average duration of immobility. The values are mean ± SD; ^∗^a significant difference *p* < 0.05, ^∗∗^a significant difference *p* < 0.01.

The results obtained in the tail suspension test confirmed that the applied protocols significantly altered depressive levels by means of the latency to the first immobility (Figure 2(a), df = 6, *F* = 6.731). The chronic oral intake of nano-HA and nano-ACP significantly reduced the latency to the first immobility (*p* < 0.01 and *p* < 0.05, respectively). However, this prodepressant action of both nano-CaPs was successfully prevented by the antioxidant supplementation with FU extract that diminished the decline in the latency to the first immobility for both nano-HA and nano-ACP compared to control and even (for nano-ACP) when compared to the group where CaP was administered solely (*p* < 0.05). At the same time, neither of the applied protocols had significantly influenced the number of immobility episodes (*F* = 2.524, Figure 2(b)). As shown in Figure 2(c), the individual application of nano-CaPs produced a different impact on the total duration of immobility (*F* = 7.031). While nano-HA and nano-ACP significantly increased this main marker of depression in TST, compared to control (*p* < 0.01 and *p* < 0.05, respectively) and even to nano-TCP (for nano-HA, *p* < 0.05), this prodepressant action was not observed in the nano-TCP group. At the same time, the restoration of the total duration of immobility was observed following simultaneous administration of FU extract in both nano-HA and nano-ACP that resulted in significant increase of this parameter (*p* < 0.05 and *p* < 0.01, respectively). Finally, the applied protocols produced no significant alterations in the average duration of immobility episodes (*F* = 0.925, Figure 2(d)).

**Figure 3:**
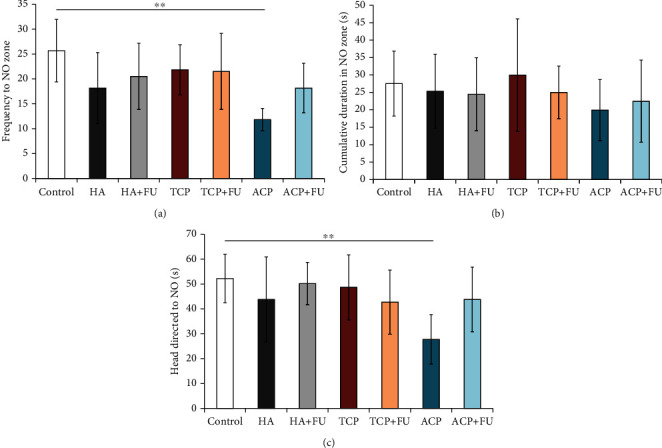
Parameters obtained in the novel object recognition test: (a) the frequency to the NO zone, (b) the cumulative duration in the NO zone, and (c) the head directed to NO. The values are mean ± SD; ^∗∗^a significant difference *p* < 0.01.

Unlike the depressive level estimation, the results of the novel object recognition test showed that long-term application of nano-CaPs led to changes in the frequency to the NO zone (df = 6, *F* = 3.123) strongly depending on nano-CaP particle structure. Thus, in the ACP group, the frequency was reduced compared to the control group (*p* < 0.01), with no changes in other groups (Figure 3(a)). As shown in Figure 3(b), none of the applied protocols significantly altered the cumulative duration in the NO zone (*F* = 0.529). However, the prolonged administration of nano-ACP significantly decreased the time interval with head directed to NO (*F* = 2.578, *p* < 0.01), with no difference observed following other protocols (Figure 3(c)). The beneficial effect of antioxidant supplementation with FU extract in the prevention of nano-ACP-induced cognitive impairment was manifested by reversing the values of spatial memory markers (Figures 3(a) and 3(c)) back to control.

**Figure 4:**
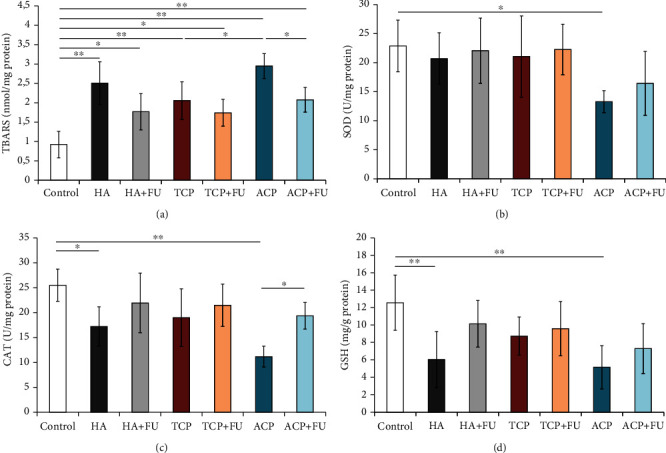
Oxidative stress parameters in rat PFC: (a) thiobarbituric acid reactive substances (TBARS), (b) superoxide dismutase (SOD), (c) catalase (CAT), and (d) glutathione (GSH). The values are mean ± SD; ^∗^a significant difference *p* < 0.05, ^∗∗^a significant difference *p* < 0.01.

As shown in Figure 4, the prolonged intake of nano-CaPs had a significant impact on oxidative stress markers in the rat PFC. All three nanosized CaPs applied in this study significantly increased the index of lipid peroxidation (Figure 4(a)), expressed as TBARS (*F* = 14.283, *p* < 0.01). Although the administration of FU extract along with CaPs reduced lipid peroxidation when compared to the groups with individual CaP intake (significant only for nano-ACP, *p* < 0.05), the TBARS values remained significantly above the control values (*p* < 0.05 for nano-HA and TCP, *p* < 0.01 for nano-ACP). The activity of antioxidant enzymes, expressed by means of SOD (Figure 4(b)) and CAT (Figure 4(c)), was significantly diminished following prolonged nano-CaP intake (*F* = 3.115 and 6.777, respectively). While the SOD activity in PFC was significantly reduced (*p* < 0.05) only in the nano-ACP group and successfully reversed with the simultaneous FU extract administration, the decline in CAT activity was observed in both nano-HA and ACP groups (*p* < 0.05 and *p* < 0.01, respectively). Again, the antioxidant supplementation restored CAT activity (*p* < 0.05). The nonenzymatic antioxidant capacity, expressed by means of GSH, in PFC (Figure 4(d)) was also diminished by nano-HA and ACP particle intake (*F* = 4.853, *p* < 0.01). The simultaneous administration of FU extract levelled GSH content up to control values.

**Figure 5:**
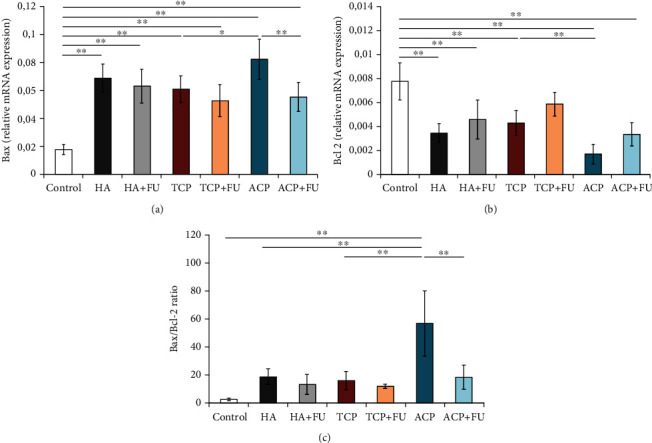
The relative gene expression of the pro- and antiapoptotic genes in the rat PFC: (a) Bax, (b) Bcl-2, and (c) Bax/Bcl-2 ratio. The values are mean ± SD; ^∗^a significant difference *p* < 0.05, ^∗∗^ a significant difference *p* < 0.01.

The evaluation of apoptotic markers (Figure 5) showed the significant alterations in both proapoptotic and antiapoptotic indicators in the rat PFC following the applied protocols. Bax relative mRNA expression was significantly enhanced by all three applied nano-CaPs (*F* = 20.963, *p* < 0.01). However, the strongest proapoptotic effect was observed in the ACP group resulting in Bax relative mRNA expression that was even significantly above the values observed in the TCP group (*p* < 0.05). On the other hand, the antioxidant supplementation with FU extract failed to prevent the proapoptotic action of the applied CaPs. The antiapoptotic mechanism, expressed by Bcl-2 relative mRNA expression, was also significantly affected by the applied protocols (*F* = 17.234). Again, all three applied CaPs significantly reduced relative Bcl-2 expression (*p* < 0.01), with the most prominent response to ACP (significant decline even when compared to the TCP group, *p* < 0.01). Like for Bax, FU extract was insufficient to prevent the diminishing effect of the applied CaPs on Bcl-2. Finally, the analysis of the Bax/Bcl-2 ratio confirmed the strong proapoptotic impact of the applied CaPs (*F* = 16.939), although not significant in the TCP group. Evidently, the strongest proapoptotic action was observed in the ACP group, but also successfully prevented by the simultaneous administration of FU extract (*p* < 0.01).

**Figure 6:**
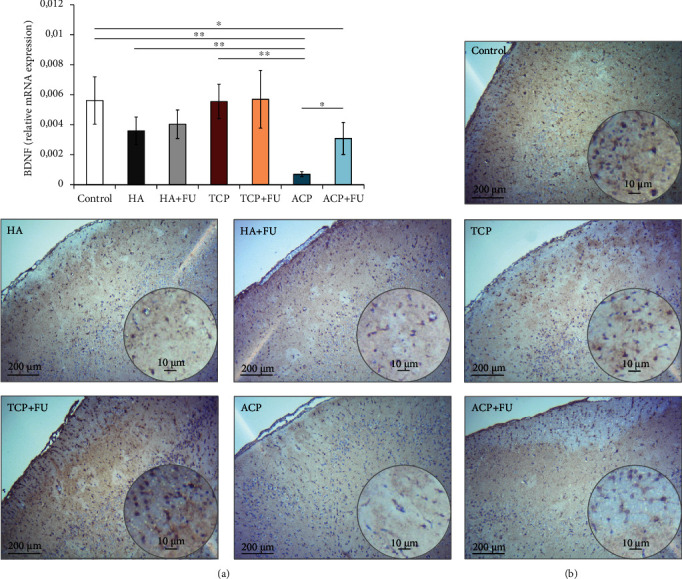
The relative gene expression and the immunohistochemical expression of BDNF in rat PFC: (a) the relative BDNF gene expression and (b) representative images of BDNF IHC staining on paraffin-embedded PFC sections (original magnification 10x and 40x; scale bar = 200 and 10 *μ*m). The values are mean ± SD; ^∗^a significant difference *p* < 0.05, ^∗∗^a significant difference *p* < 0.01.

The RT-PCR analysis of PFC tissue homogenate (Figure 6) showed the significant alterations in BDNF mRNA relative expression (*F* = 13.246), manifested by significant decline achieved only in the ACP groups (*p* < 0.01), while the other two nanosized CaPs did not induce significant decrease. Although the simultaneous administration of FU extract resulted in a significant increase in BDNF expression, compared with the group where ACP was applied solely (*p* < 0.05), the BDNF levels in the combined group remained significantly below the control values (*p* < 0.05).

**Figure 7:**
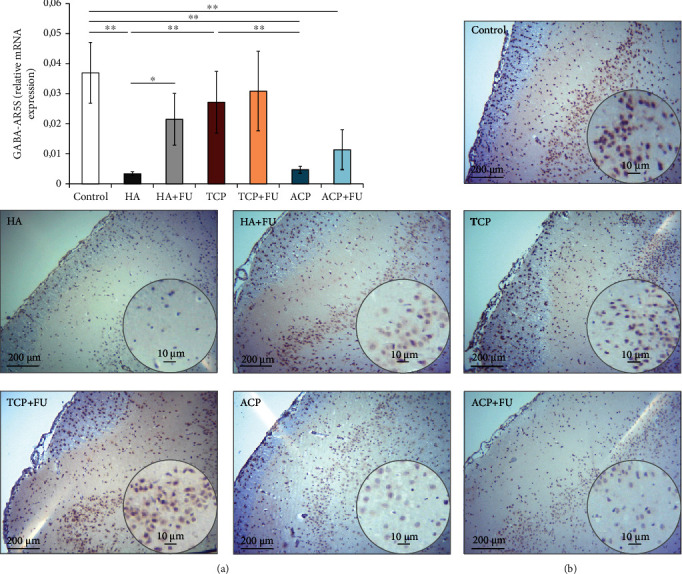
The relative gene expression and the immunohistochemical expression of the GABA-AR5S in rat PFC: (a) the relative GABA-AR5S gene expression and (b) representative images of GABA-AR5S IHC staining on paraffin-embedded PFC sections (original magnification 10x and 40x; scale bar = 200 and 10 *μ*m). The values are mean ± SD; ^∗^a significant difference *p* < 0.05, ^∗∗^a significant difference *p* < 0.01.

The expression of GABA-A receptors in PFC, estimated by means of subunit 5 relative mRNA expression, as shown in Figure 7, was also significantly altered by the applied protocols (*F* = 14.471). Nano-HA and ACP particles significantly decreased GABA-AR expression (*p* < 0.01), while TCP-induced decline was not significant. Interestingly, the reduction of GABA-AR5S observed in the HA and ACP groups was significant even when compared to the TCP group (*p* < 0.05 and *p* < 0.01, respectively). The antioxidant supplementation with FU extract significantly restored GABA-AR5S expression in the HA group (*p* < 0.05) but failed to restore GABA-AR expression in the ACP group that remained significantly below the control values (*p* < 0.01).

## 4. Discussion

Increasing interest in the application of bone replacement materials, as expected, is accompanied by the necessity for the estimation of the adverse effects of such a therapeutic approach in a broad field of medical disciplines, most frequently in dentistry. However, since there is evidence that CaPs, as main dental tissue remineralizing agents, are found to cross the blood-brain barrier as nanoparticles [26], there is still a need to evaluate the impact of those compounds on the central nervous system following prolonged systemic administration, in order to mimic clinical conditions for their usage. It should be emphasized that this study was performed with the minimal doses of nano-CaPs, corresponding to the previous investigations that analyzed the expected release of CaPs [28], and also to the minimal doses for the systemic application that was reported to produce toxic effects in other organ systems [27]. Furthermore, the individual doses for nano-Ca compounds applied in this study were set to be equimolar in order to allow the comparison of the observed outcome, based on predefined nano-HA dose for parenteral usage [27] and nano-ACP dose for the release capacity from dental composites [28], as referent values. Also, the daily dose of FU extract was chosen, by means of optimal biological effectiveness, according to our previous findings [18, 20].

The results of our study strongly confirmed the oxidative damage induced by nanosized CaP particles. However, the response in the oxidative stress markers significantly differed depending on the composite type. While the lipid peroxidation was enhanced by all three applied CaPs, the antioxidant system, expressed by means of both enzymatic and nonenzymatic capacity, was not disturbed by all applied substances. Thus, with no significant effect of nano-TCP, nano-HA mostly affected CAT and GSH, but nano-ACP induced the total reduction of the evaluated antioxidant mechanisms (SOD, CAT, and GSH, Figure 4). Our results are in line with the reported prooxidative action of HA nanoparticles *in vitro*, where their application increased ROS levels and decreased SOD and GSH in the osteoblastic MC3T3-E1 [11] and C6 cells [40]. However, there is no data considering the effects of TCP and ACP. Since there is no literature data for the impact of CaPs (in nanoparticles) on the oxidative stress markers *in vivo*, we can only compare the observed alterations with other nanosized metallic particles. Thus, the results of our study are in line to previously reported oxidative damage in the brain (with no focus on PFC) following ZnO [41], CuO [42], Ag [43], and TiO_2_ [44] administration.

The CaP-induced oxidative damage was significantly attenuated with the simultaneous administration of FU extract. Thus, the antioxidant supplementation along with CaP particles prevented the decline in the activity of the antioxidant enzymes, as well as GSH content in PFC. At the same time, FU extract decreased lipid peroxidation, but the values remained above control (Figure 4). Again, due to the lack of literature data, we cannot compare our results with the estimation of the antioxidant supplementation impact on CaP nanoparticle-induced oxidative damage. Instead, we can only comment that amelioration of metallic nanoparticles (ZnO, CuO, and Ag) prooxidative action in the brain tissue was achieved with different phytochemical compounds such as flavonoid glucosides hesperidin [45] and rutin [43], as well as crocetin [46], a diterpenoid from saffron spice.

As numerous literature data stated that the oxidative imbalance may often be accompanied by the apoptosis-mediating pathways [47], we also estimated the impact of the prolonged nanosized CaP intake on the apoptotic markers in PFC. Indeed, the results obtained in this study confirmed the proapoptotic action of all investigated nanosized CaPs that was manifested by both upregulation of the proapoptotic (Bax) and downregulation of the antiapoptotic (Bcl-2) markers (Figure 5). However, it should be noticed that the proapoptotic impact in the nano-ACP-treated group was more pronounced than for HA and TCP, and this proapoptotic action of ACP, although significantly reduced by FU extract, remained markedly above the control values. The results obtained in both *in vitro* and *in vivo* studies also confirmed the stimulation of proapoptotic mechanisms following the nanosized CaP administration. It has been reported that HA nanoparticles increased apoptosis in osteoblasts and macrophages [48, 49] by the mechanism that involves increased p53 expression and caspase family activity with the simultaneous downregulation of Bcl-2, while ACP nanoparticles induced apoptosis of leukemia cells by selective effect in G1 phase [14]. In order to compare the proapoptotic action of nanosized CaPs as observed in this study, it should be mentioned that the other metallic nanosized compounds such as CuO [46] also enhanced apoptosis in hippocampal HT22 cells by Bax upregulation and Bcl-2 downregulation. Furthermore, *in vivo* studies that evaluated the influence of the nanosized CaPs on apoptosis were conducted only with HA and TCP, but with no specific data for the brain tissue. Nano-HA particles induced morphologically verified apoptosis in the rat liver and kidney [27], and this proapoptotic action of nano-HA was accompanied by an increase of Bax expression [50]. Nano-*β*-TCP induced apoptosis of tumor cells by the apoptotic signalling that resulted in overexpression of Bax, caspase-3, and caspase-9 [13]. Like for the oxidative damage, the beneficial effects of antioxidant supplementation on the nanosized CaP-induced apoptosis were not evaluated in the brain tissue, but were confirmed with medicinal plant chicory (*Cichorium intybus*) [50] and pure compounds curcumin and chitosan [17] in the rat kidney.

The analysis of the relative mRNA expression in PFC showed a significant decline for both BDNF and GABA-AR, expressed by subunit 5, following the prolonged intake of HA and ACP, with no significant alterations observed in the TCP group (Figures 6 and 7, respectively). Also, our results showed that simultaneous antioxidant supplementation with FU extract attenuated the decline in BDNF and GABA-AR5S expression, although the values in the ACP+FU group remained significantly below the values observed in the control group. Since there is no literature data for the influence of nanosized CaP particles on BDNF and gabaergic system in the brain tissue, we can only compare our results with the influence of silver nanoparticles. Thus, it was shown that the silver nanoparticles produced a significant decrease in various monoamine neurotransmitters, including GABA, levels in the rat brain homogenate [43] and also inhibited BDNF signalling in the human neuroblastoma cell line SH-SY5Y [51]. It is worth to notice that the antioxidant supplementation with rutin prevented the decline in monoamine neurotransmitter levels [43].

Taking into account previously commented results, it is not surprising that both depressive state levels and cognitive functions estimated in this study were significantly affected by the chronic CaP nanoparticles intake (Figures 2 and 3). Interestingly, the prodepressant action was observed within both HA and ACP groups, while a significant cognitive impairment was noticed only in the ACP group. However, it seems important to underlie that both investigated behavioural patterns were successfully prevented by the simultaneous antioxidant supplementation with FU extract. As already declared, due to the lack of data for nano-CaP particles' impact on those specific behavioural patterns, we can only compare the behavioural alterations observed in this study with the behavioural impact of some other nanosized metallic compounds. Therefore, the most frequently investigated metallic compound, nano-TiO_2_, produced an adverse effect on rodent behaviour by means of both prodepressant action [52, 53] and cognitive impairment [52, 54]. The prodepressant action of nanosized metallic compounds was also observed with ZnO nanoparticles [55], while the disruption of learning and memory was also reported following CuO nanoparticle application [56]. However, none of the mentioned investigations employed the antioxidant supplementation to estimate the potential role of oxidative stress in the mediation of metallic nanoparticle-induced behavioural disturbances.

Finally, the observed differences between the individual CaPs applied in this study may be addressed to different Ca/P ratio [57] and compound structure—crystallization [58], which still have to be evaluated in our future investigations. However, it seems that the antioxidant supplementation (with FU extract) in this study may be useful in the prevention of the behavioural manifestations of nano-CaP-induced neurotoxicity.

## 5. Conclusions

In summary, the widespread medical use of nano-CaPs is accompanied by the confirmed toxicities, so it requires further elucidation of a general mechanism that may compromise the efficiency and safety. According to the results obtained in this study, it appears that the antioxidant supplementation may prevent the processes underlying the side effects of nano-CaPs, including the neurotoxicity manifested through the specific behavioural alterations.

## Data Availability

Data are available upon request.

## Supplementary Materials

Supplementary Materials Supplementary file 1: list of chemicals for oxidative stress determination. Supplementary file 2: list of primers used for RT-PCR analysis.
