# Supplementary material for: The Beneficial Role of Filipendula ulmaria Extract in Prevention of Prodepressant Effect and Cognitive Impairment Induced by Nanoparticles of Calcium Phosphates in Rats
Source: Oxid Med Cell Longev. 2021 Feb 10;2021:6670135. doi: 10.1155/2021/6670135 (PMC7895592; doi:10.1155/2021/6670135)
Supplement: Supplementary Materials — Supplementary file 1: list of chemicals for oxidative stress determination. Supplementary file 2: list of primers used for RT-PCR analysis. [file 6670135.f1.docx]

**Supplementary files**

**Supplementary file 1.** List of chemicals for oxidative stress determination

- Methanol (*pro analysis*, min 99.8%) was obtained from MOSS (Belgrade, Serbia).
- Bovine serum albumin (BSA) was purchased from Human GmbH (Wiesbaden, Germany).
- L-Glutatione reduced, (−)-epinephrine, sodium dodecyl sulfate (SDS) and 5,5′-dithiobis (2-nitrobenzoic acid) (DTNB) were purchased from Sigma-Aldrich Chemie GmbH (Darmstadt, Germany).
- 2-Thiobarbituric acid (TBA) was obtained from ABCR GmbH (Karlsruhe, Germany).
- 1,1,3,3-Tetraethoxypropane (malonaldehyde-bis(diethyl acetal), MDA) was purchased from Acros Organics (Geel, Belgium).

**Supplementary file 2.** List of primers used for RT PCR analysis

|  | **foward** | **reverse** |
| --- | --- | --- |
| **β-actin** | AAGATCCTGACCGAGCGTGG | CAGCACTGTGTTGGCATAGAGG |
| **Bax** | CGGCGAATTGGAGATGAACTGG | CTAGCAAAGTAGAAGAGGGCAACC |
| **Bcl-2** | TGTGGATGACTGACTACCTGAACC | CAGCCAGGAGAAATCAAACAGAGG |
| **BDNF** | AGCTGAGCGTGTGTGACAGT | ACCCATGGGATTACACTTGG |
| **Gabra5** | CCCTCCTTGTCTTCTGTATTTCC | TGATGTTGTCATTGGTCTCGTCT |
